# Carbon Dioxide Euthanasia Selectively Affects Physiology of Murine Retinal Cells, Implicating Carbonic Anhydrase-Expressing Cell Populations

**DOI:** 10.1167/iovs.67.3.61

**Published:** 2026-03-31

**Authors:** Irina Ignatova, Ari Koskelainen

**Affiliations:** 1 Department of Neuroscience and Biomedical Engineering, School of Science, Aalto University, Finland

**Keywords:** carbon dioxide, euthanasia, mice, retina, electroretinography

## Abstract

**Purpose:**

Laboratory rodents are commonly euthanized by exposure to gradually increasing concentrations of carbon dioxide (CO_2_). CO_2_ exposure induces respiratory acidosis, reduces dopamine levels, and causes hypoxia in central nervous system tissues, potentially affecting their physiology. These effects may be critical for brain and retinal tissues, yet the impact of CO_2_ euthanasia remains largely unclear.

**Methods:**

Using dark-adapted transretinal electroretinography (tERG), we tested the hypothesis that terminal CO_2_ overdose alters mouse retinal physiology. Two CO_2_ displacement rates were used, 30% and 60% of the chamber volume/min, with cervical dislocation as a reference method.

**Results:**

Neither slow nor fast CO_2_ overdose euthanasia affects rod photoreceptor and ON-bipolar cell flash responses. Activation and deactivation of rod phototransduction were not affected by CO_2_ overdose. However, both flow rates of CO_2_ exposure led to decreased cone photoreceptor response amplitudes and increased power spectral density integrals of oscillatory potentials (OPs). Moreover, Müller glia flash response amplitudes were reduced, and OPs were faster and more synchronized with the slower CO_2_ displacement rate compared to the two other euthanasia methods. In the mammalian retina, carbonic anhydrase is expressed in Müller glia, retinal pigment epithelium, most cone photoreceptors and a subset of amacrine cells.

**Conclusions:**

Our findings indicate that CO_2_ euthanasia can generally be considered a safe termination method for retinal research, but caution should be taken when studying the physiology of carbonic anhydrase-expressing cells.

Euthanasia of laboratory animals should be performed as painlessly and efficiently as possible. The most common methods for adult mice euthanasia are carbon dioxide (CO_2_) overdose, cervical dislocation, and overdose of anesthetics.^1^

Carbon dioxide inhalation is widely used for euthanizing laboratory rodents because of its association with anesthetic depth and reduced pain sensitivity via cerebrospinal fluid acidification.^2^ Guidelines from the American Veterinary Medical Association recommend gradual termination chamber filling for CO_2_ euthanasia and the EU Directive 2010/63/EU mandates gradual filling. Gradual filling is used to minimize pain by ensuring animals lose consciousness before reaching painful CO_2_ concentrations. Pain from CO_2_ exposure arises when the gas reacts with moisture on the respiratory, oral, nasal, and ocular mucosa to produce carbonic acid. Most nociceptors in humans and animals begin responding at CO_2_ levels exceeding ∼37%.^3^^,^^4^

The appropriate CO_2_ displacement rate for gradual filling remains a topic of ongoing debate.^5^^–^^9^ Current U.S. guidelines recommend filling the chamber with CO_2_ at a rate of 30%–70% of its volume per minute,^10^ whereas from 2013 the recommended rate was 10%–30%.^11^ In Europe, there is no official recommendation for CO_2_ flow rates,^1^ but 20%–40% per minute is commonly used in animal facilities.

Beyond pain, CO_2_ inhalation can induce distress by triggering a sensation of breathlessness (“air hunger”) and anxiety behavior through activation of ion channels in the amygdala linked to fear responses.^9^^,^^12^^,^^13^ Some physiological and behavioral studies suggest that slow CO_2_ fill rates prolong distress before the rodent loses consciousness, without clear evidence of pain reduction compared with faster CO_2_ fill rates.^7^^,^^8^^,^^14^ However, another study reported increased anxiety-like behaviors in mice euthanized using fast CO_2_ fill rates (70%/min) compared with slow (30%/min).^5^ Therefore a tradeoff must be reached between minimizing pain, dyspnea, and fear behaviors during CO_2_ euthanasia.

Beyond these antemortem effects of CO_2_, the euthanasia method can significantly influence postmortem physiological measurements and potentially alter experimental results.^6^^,^^15^ Specifically, CO_2_ euthanasia induces respiratory acidosis in mice, evidenced by a blood pH of 6.9 ± 0.1 compared with 7.2 ± 0.1 after ketamine–xylazine euthanasia.^16^ Immediately after death, CO_2_ overdose leads to significantly lower brain tissue pH (6.0 ± 0.02) relative to decapitation or barbiturate overdose (pH 6.8 ± 0.04), with no recovery within five minutes after death.^17^ Furthermore, mice euthanized via CO_2_ overdose show a substantial decrease in retinal dopamine,^18^ which serves as a neuromodulator in both the retina and brain. Another study reported that CO_2_ exposure induces changes in the mechanical properties of brain tissue, indicating underlying chemical alterations; specifically, stiffness increased in gray matter, while white matter remained unaffected.^17^ Moreover, CO_2_ displaces oxygen in inspired air, resulting in hypoxia. Brain and retinal tissues, which are highly sensitive to oxygen deprivation and other environmental changes, are potentially the most vulnerable to the effects of CO_2_-induced euthanasia. Despite limited understanding of how CO_2_ euthanasia affects brain and retinal physiology, many neurobiology laboratories routinely use CO_2_ to euthanize laboratory animals.

Using transretinal electroretinography (tERG), we tested the hypothesis that terminal CO_2_ overdose alters retinal functioning. To identify potential targets, we isolated responses from rod and cone photoreceptor cells, ON-bipolar cells, and Müller glia, as well as oscillatory potentials mainly mediated by amacrine cells. We investigated two chamber volume displacement rates of CO_2_: slow (30%/min) and fast (60%/min). Cervical dislocation was used as the reference method.

Our results demonstrate that CO_2_ overdose euthanasia does not significantly affect rod phototransduction or signal transmission from rods to ON-bipolar cells. However, the slower displacement rate of CO_2_ decreases response amplitudes from Müller glia and accelerates and synchronizes oscillatory potentials, while both slow and fast CO_2_ overdose increase the power spectral density (PSD) integral of oscillatory potentials (OPs) and decrease the response amplitude from cone photoreceptor cells. Thus, when applying CO_2_ overdose euthanasia, we recommend using a fast CO_2_ displacement rate and avoiding CO_2_ euthanasia when investigating the physiological properties of carbonic anhydrase–expressing cells.

## Methods

### Animals and Ethical Approval

C57BL/6JRccHsd wild-type mice (*Mus musculus*) were bred locally at Laboratory Animal Centre, Biomedicum, University of Helsinki and maintained under a light-dark cycle (12-hour light and 12-hour darkness) with free access to food and water. Mice of both sexes, aged 2.7–13.4 months were used (for the three study groups, medians were 3.1–3.3 months and the contribution of females in each group was 46% to 54%). Some tERG recordings were conducted during the animals’ subjective night; the contribution of such experiments in each study group was 31% to 38%. Mice were dark-adapted overnight before experiments.

Animals were sacrificed under dim far red LED illumination (peak wavelength 690 nm; LED690-03AU; Roithner Lasertechnik GmbH, Vienna, Austria) using cervical dislocation or CO_2_ overdose. Cervical dislocation was performed by placing a screwdriver at the base of the skull and quickly pulling the base of the tail, resulting in the separation of the cervical vertebrae from the skull. The residual heartbeat was monitored by palpation, and death was confirmed several minutes after cervical dislocation.

CO_2_ overdose was carried out by placing the mouse in a transparent cubic chamber (not prefilled) and gradually filling it with 100% CO_2_ at a rate of 30% or 60% of the chamber volume per minute. Volumetric flow rate was kept at a constant level using a manual bubble flowmeter. The CO_2_ flow was maintained for ∼2.5 minutes (60% flow rate) or ∼4 minutes (30% flow rate) until the cessation of breathing and then for an additional one minute. The mouse remained in the chamber for a further five minutes to ensure death was achieved, in accordance with the local facility's protocol. All experiments were conducted according to the Legislation on animal experiments in Finland and to the ARVO Statement for the Use of Animals in Ophthalmic and Vision Research.

### Transretinal Electroretinography

The specimen holder was 3D-printed from biocompatible BioMed clear resin (FormLabs, Somerville, MA, USA) on a Formlabs Form 2 resin printer. To enhance the holder's transparency, the translucent resin above the retina was replaced with a borosilicate glass attached with biocompatible epoxy adhesive (Loctite M-31 CL; Henkel Corporation, Düsseldorf, Germany). The specimen holder consists of top and bottom parts, each containing tunnels for Ag/AgCl pellet electrodes (EP2; World Precision Instruments, Sarasota, FL, USA), whereas only the top part has a tunnel for perfusion. The bottom part includes a dome with a 1.75 mm-diameter vertical tunnel (recording area), onto which the tissue is placed on top of a polycarbonate membrane (5 µm pore size). The tunnels were filled with a solution containing (in mM): 140 NaCl, 3.6 KCl, 2.4 MgCl_2_, 1.2 CaCl_2_, 3 HEPES, 0.01 EDTA (pH set to 7.4 with NaOH) to ensure stable electrical contact with the two Ag/AgCl pellet electrodes (trans-electrode resistance was 6–7 k*Ω*).

After euthanasia, eyes were enucleated, and dissection was performed at room temperature in bicarbonate-buffered Ames’ medium (see below) bubbled with carbogen (pH ∼7.3) under dim far red illumination (660 nm LED with 665 nm long-pass filter; Thorlabs, Newton, NJ, USA). One isolated retina was placed in the specimen holder photoreceptor side upwards. The retina was continuously perfused (∼5.5 mL/min) with Ames’ medium with L-glutamine (no. A1372-25; United States Biological, Salem, MA, USA) supplemented with 22.6 mM of NaHCO_3_ and penicillin-streptomycin antibiotic mix (6 mL/L of solution; Sigma-Aldrich, St. Louis, MO, USA). The solution was pre-heated to ∼32.5°C–33.5°C using the inline heater SH-27B controlled by a TC-324B temperature controller (Warner Instruments, Hamden, CT, USA) and bubbled with carbogen through a 25–50 µm porosity gas dispersion tube (Sigma-Aldrich) to maintain a pH of ∼7.45. When needed, 100 µM of BaCl_2_ (Sigma-Aldrich) and 40 µM of DL-2-Amino-4-phosphonobutyric acid (DL-AP4, ab120001; Abcam, Cambridge, UK) were used to block the Müller glial component and the synaptic transmission from photoreceptors to ON-bipolar cells, respectively. Although both retinas were isolated during dissection, recordings were performed on one retina at a time. The second retina was stored at room temperature in bicarbonate-buffered Ames’ medium inside a light-proof container, continuously bubbled with carbogen (pH ∼7.3).

Signals were amplified 10^4^-fold, low-pass filtered at 1 or 10 kHz (second-order Bessel filter) using a differential amplifier (model 3000; A-M Systems, Sequim, WA, USA) and recorded at a sampling frequency of 4 kHz using PCIe-6351 (National Instruments, Austin, TX, USA) DAQ-board and custom-made MATLAB-based data acquisition software (MathWorks, Natick, MA, USA).

### Light Stimulation

Dark-adapted retinas were stimulated using a fiber-coupled green LED (peak wavelength 530 nm, M530F2; ThorLabs) with 515 nm long-pass filter (OG515; Edmund Optics, Barrington, NJ, USA) connected to a LED driver (LEDD1B; ThorLabs) and controlled through the data acquisition software. Light intensity was attenuated with a series of absorptive neutral density filters (from ND5 to ND1 that correspond to the transmission values from 10^−5^ to 10^−1^ from the original intensity). Flashes of 4 ms duration (2 ms for cones in the double-flash protocol) were delivered in twofold intensity increments. Interstimulus interval was 10 seconds for dim, 20 seconds for sub-saturating, and 40 seconds for saturating flashes.

The stimulus light strength was calibrated using a photodiode connected to an optical power meter (S121C and PM100D; ThorLabs). The measured optical power was converted to the number of photoisomerized visual pigment molecules, estimated for the retina illuminated from the photoreceptor side as described previously.^19^ Loss in the light transmission through the recording chamber was accounted for during calibration (optical transmittance of borosilicate glass is ∼90% in the visible light range).

The number of rhodopsin photoisomerizations in rod cells (R*/rod) was calculated using the visual pigment template,^20^ incorporating the collecting area of the mouse rod, the LED emission spectrum, transmittance of OG515, and the photodiode spectral sensitivity and optical power meter's sensitivity. The end-on collecting area of the rod was calculated using the following parameters: an outer segment diameter of 1.4 µm, length of 24 µm, optical density of 0.016 µm⁻¹, and a quantum efficiency of 2/3.^19^ The collecting area was calculated to be 0.6 µm² at a peak of rhodopsin absorption and 0.47 µm² at 530 nm.

The variable proportion of M-opsin expression in different mouse cones, along with potential shadowing of cones by rod outer segments, introduces uncertainty when estimating the effective collecting area of cone photoreceptors.^19^ Therefore in this study we did not convert the flash power stimulating cones into M-opsin photoisomerizations.

### Data Analysis

Data analysis was performed using custom-made MATLAB-based scripts. The original tERG response contains low-frequency signal components and higher-frequency oscillatory potentials (OPs). Using distinct signal processing approaches, we separated OPs and low-frequency tERG signal as follows.

**Figure 1.**
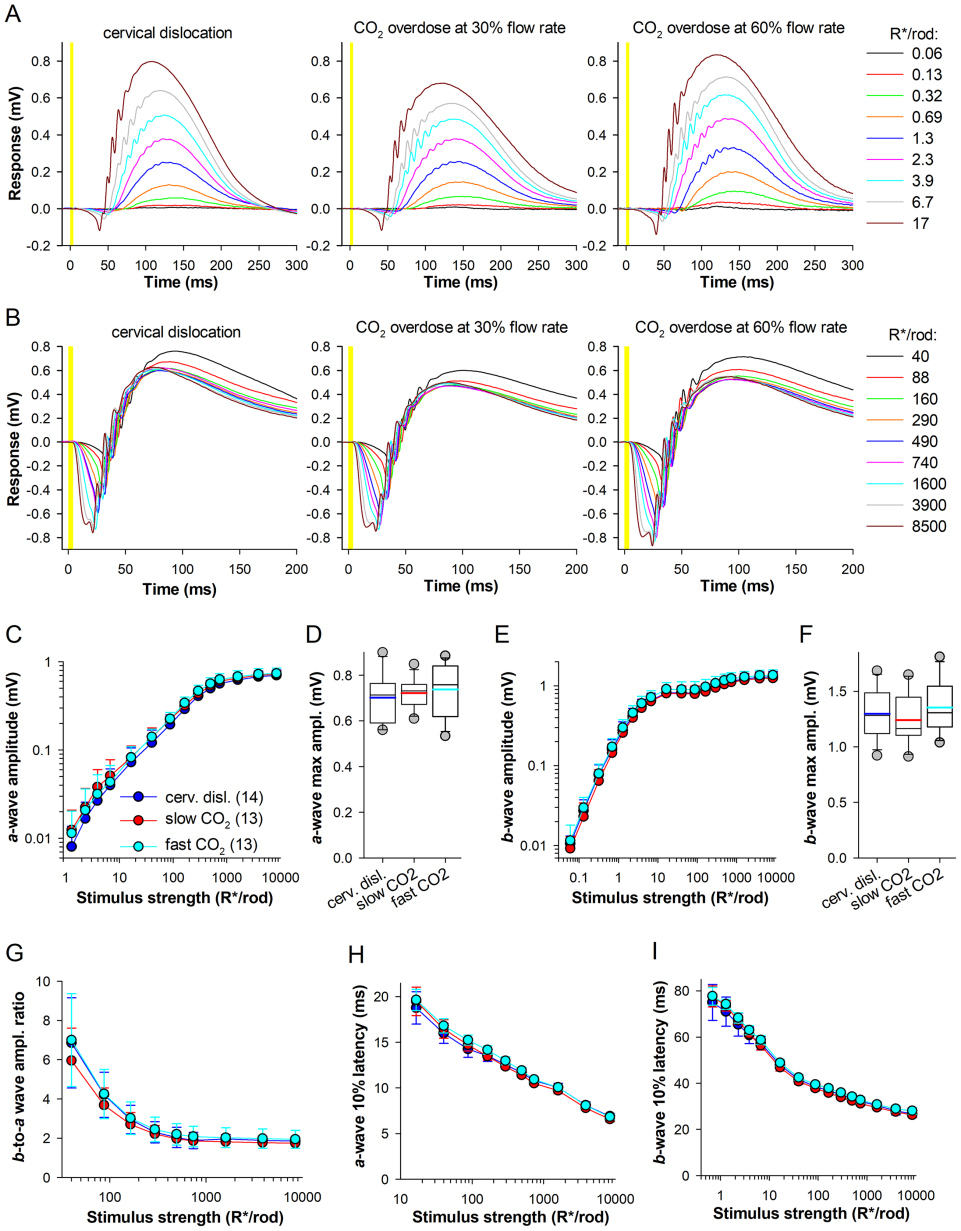
**Dark-adapted tERG *a*-and *b*-waves.** **(A**, **B)** Representative tERG responses from WT mice euthanized by cervical dislocation (*left*) and slow (*center*) and fast CO_2_ overdose (*right*) for the dimmer **(A)** and brighter **(B)** flashes; here and elsewhere, 4 ms-long flashes were given starting at 0 ms (marked with *yellow shading*). **(C, E)** Dependencies of *a*-wave and *b*-wave amplitudes on stimulus strength; here and elsewhere, mean ± std and (*n*) denotes the number of retinas. **(D, F)** Box plots comparing *a*-wave and *b*-wave maximal amplitude. Here and elsewhere in box plots: thin black horizontal lines denote medians and thick color lines averages; the upper and lower edges of the box are the upper (25th) and lower (75th) quartiles, respectively; whiskers indicate the 10th and 90th percentiles; outliers are individual data points (circles) outside the whisker range; adjusted *P* values were indicated if difference was significant. **(G)** Dependencies of *b*- to *a*-wave amplitude ratios on stimulus strength. **(H, I)** Dependencies of *a*-wave and *b*-wave 10% latency on stimulus strength. Experiments were performed at ∼33°C, with 100 µM Ba^2+^ present in all cases except those shown in Figure 6.

**Figure 2.**
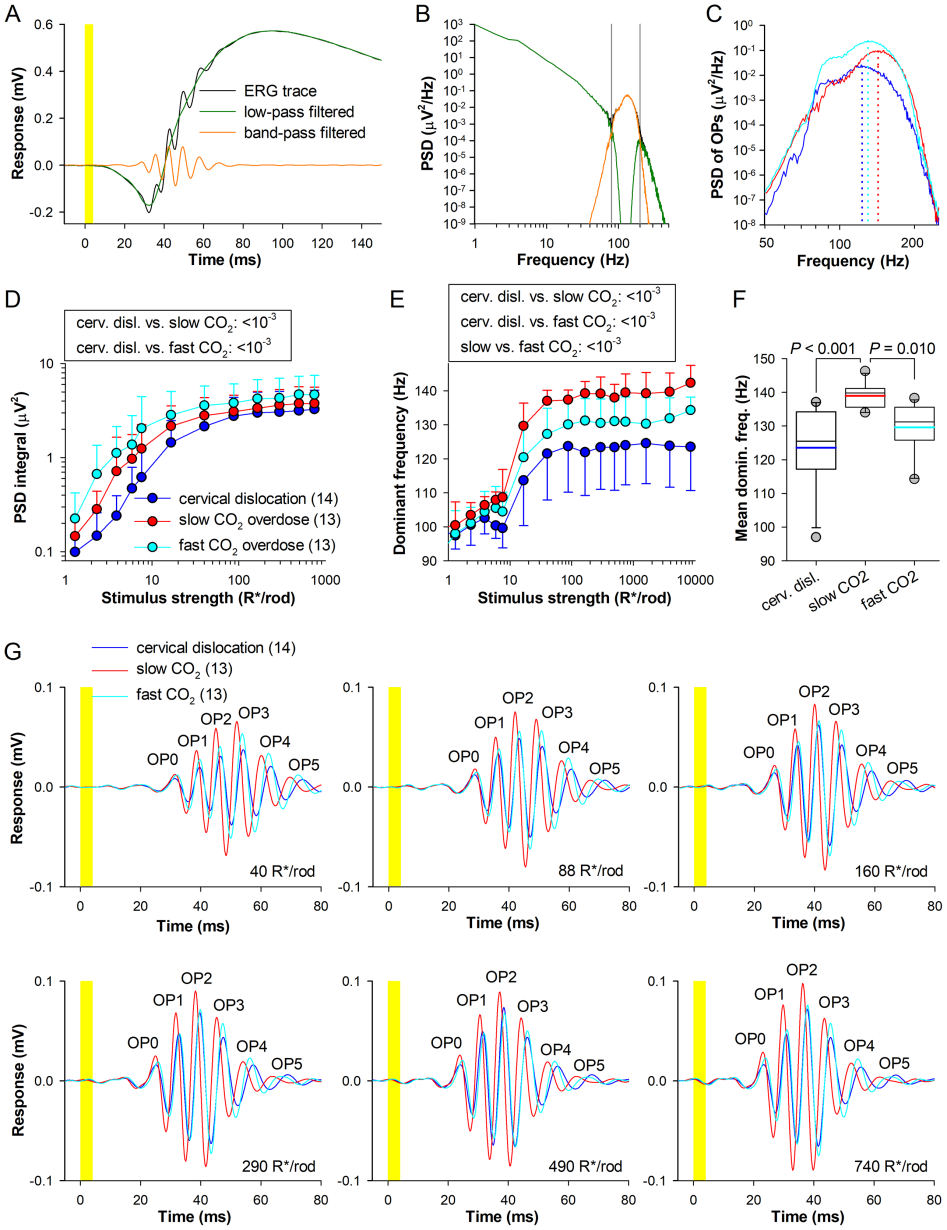
**Oscillatory potentials.** **(A)** Dark-adapted tERG waveform and the same trace filtered using band-pass filtering with a passband of 80–200 Hz and low-pass filtering with a cutoff frequency of 80 Hz (zero-phase digital filtering with fourth-order Butterworth filter). **(B)** Corresponding PSD estimated through Welch's method using one-second–long Hanning window with 50% overlap. PSD within the vertical gray lines (80–200 Hz range) corresponds to OPs. **(C)** Representative PSD traces of OPs for mice euthanized with three different methods. Stimuli were ∼160 R*/rod. **(D)** Integrals of PSD within the 80–200 Hz range, plotted against stimulus strength for the three study groups. **(E)** Dominant frequency corresponding to OPs is shown as a function of stimulus strength. **(F)** Mean dominant frequency for ≥40 R*/rod. **(G)** Group-averaged OPs for mice euthanized using three different methods for six different stimulus strengths.

For *a*- and *b*-waves/components analysis, OPs were removed by applying a low-pass filter with a cutoff frequency of 80 Hz to the tERG signals (90 Hz for the 2-3 brightest stimuli; zero-phase digital filtering with 4^th^-order Butterworth filter, Figs. 1, 2, Supplementary Fig. S1). To ensure that this low-pass filtering did not affect the photoreceptor component waveforms, 40 µM of DL-AP4 was applied to eliminate all OPs. Filtering had no effect on the remaining photoreceptor component waveforms. Two main flash response parameters were derived: amplitude and 10% latency. The amplitudes of *a*-waves were determined from the baseline to the trough, whereas the *b*-wave amplitude was measured from this minimum to the highest peak. Latency values were determined as the time interval between the flash onset and the time responses reached 10% of their maximum. For the brightest flashes activating both rod and cone pathways, the amplitude of the photoreceptor component was estimated at times after the faster cone responses.

The rising phases of the photoreceptor component were analyzed using the Lamb and Pugh activation model,^21^ which describes the kinetics of phototransduction before the onset of significant deactivation. The early phases of rod responses were fitted with the equation:

(1) $$R\left(t\right)=R_{max}\left(1-\;e^{-\;\phi \cdot A\;\cdot \frac{{\left(t-t_{d}\right)}^{2}}{2}}\right),\;\;t>t_{d}$$

where *R(t)* is the response waveform, *R_max_* the saturated response amplitude, ϕ the number of photoisomerizations per rod cell, *A* the activation constant, *t* time after the flash and *t_d_* time delay due to phototransduction reactions. Fits were restricted to the early rising phase of 3-4 sub-saturated responses (up to time point where the response reached 50% of its peak amplitude, or to 40 ms if the 50% level was reached later). For each retina, a single global activation constant *A* and a single global delay *t_d_* were obtained by nonlinear least-squares fitting (Levenberg–Marquardt algorithm) using a custom MATLAB script. The goodness of the global fits was assessed using coefficient of determination (*R^2^*) and root-mean-square error (*RMSE*), with mean values of 0.994 for *R^2^* and 0.004 for *RMSE*, indicating excellent agreement between the model and the experimental data.

**Figure 3.**
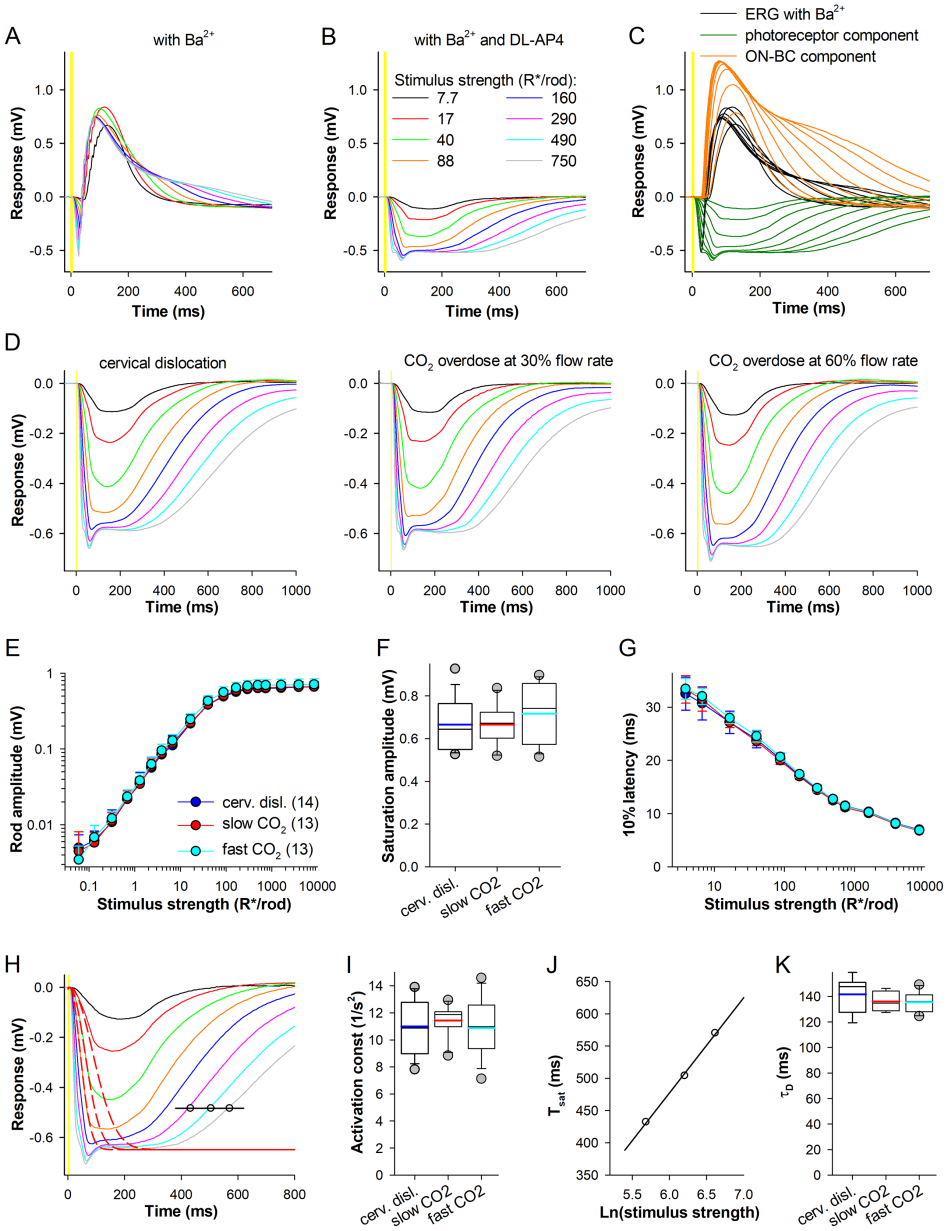
**Rod photoreceptor component of tERG.** **(A**, **B)** The tERG recordings from a mouse retina before **(A)** and during application of synaptic blocker DL-AP4 **(B)**. **C**, Subtracting the photoreceptor component **(B)** from full tERG **(A)** yields ON-bipolar cell component (*orange traces*, all traces in **C** were low-pass filtered). **(D)** Group-averaged photoreceptor component responses from mice euthanized by cervical dislocation (*left*) and by slow and fast CO_2_ overdose (*center, right*). **(E**, **F)** Stimulus-response relationships for the rod photoreceptor component **(E)** and its saturated response amplitudes **(F)** across the three groups. **(G)** The 10% latencies for the three groups as functions of stimulus strength. **(H)** Example of fitting the Lamb & Pugh activation model to a set of sub-saturated responses (*red dashed traces*). Saturated flash responses were used to determine dominant time constant, τ_*D*_ (time points *T_sat_*, where responses return to 75% level, are indicated by circles on the black horizontal line). **(I)** Activation constants for the three study groups at ∼33°C. **(J)** Pepperberg plot for the data in **H**. τ_*D*_ is determined from the linear fit (*black trace*). **(K)** τ_*D*_s for the three study groups.

The dominant time constant of recovery τ_*D*_ was estimated from the recovery phase of three saturated flash responses as described previously.^22^ The times for saturated photoresponses to recover to a criterion level (*T_sat_*), chosen as 25% from the saturated amplitude (see Fig. 3H), were plotted against the natural logarithm of flash strength (*ln*(ϕ)). The relationship is approximately linear for high-stimulus flashes:

(2) $$T_{\mathrm{sat}}\left(\phi \right)=\tau_{D}\cdot ln\left(\phi \right)+\mathrm{const}$$

Linear regression of *T_sat_* versus *ln*(ϕ) was used to determine τ_*D*_, representing the rate-limiting step in recovery from saturation. The goodness of the fits was evaluated using *R^2^*, with mean value of 0.988.

Saturated response amplitude and half-maximal light power for cone photoreceptors were estimated from fitting the Michaelis equation using the least-squares parameter method:

(3) $$R\left(P\right)=R_{max}\frac{P}{P+\;P_{\frac{1}{2}}}$$

where *R* is the response amplitude, *R_max_* the saturated response amplitude, *P* the light power, and *P_1/2_* the light power producing half-maximal response.

OPs were isolated using band-pass filter with stopband of 80–200 Hz (zero-phase digital filtering with 4^th^-order Butterworth filter, Figs. 2A, 2B, Supplementary Fig. S1). For the two brightest stimuli, lower cutoff frequency was increased (up to ∼110 Hz). The PSD was calculated using Welch's method with a 1s-long Hanning window and 50% overlap.

The local extrema of isolated wavelets were detected using a certain threshold amplitude (∼10% from the maximal peak). While OP0 was clearly observed in all recordings, we kept the “traditional” OP numbering so that OP2 and OP3 exhibit the highest oscillatory potential amplitude (Fig. 2G). Two main parameters were derived from individual OPs: amplitude and time-to-peak. Amplitude of OPs were measured from the trough to the peak of the corresponding wavelet. Time-to-peak denotes the time taken to reach the maximum positive amplitude of each wavelet.

### Statistics

At the initial stage of statistical analysis, the Shapiro–Wilk normality test and equal variance test was used to determine if data could be analysed using parametric statistical methods. The samples that passed these tests were presented as mean ± standard deviation (std) and analysed using one-way ANOVA followed by Holm-Sidak post hoc test.

Data in the samples that violates parametric assumptions of normality or equal variances were compared using Kruskal–Wallis test followed by Dunn's test; in the text, such data were presented as medians and interquartile range (25% quartile–75% quartile).

For evaluation of test parameters across a range of stimulus strengths, data were pooled within the near-linear range of the stimulus–response relationship (on either a log–log scale or linear–log scale). The logarithm of stimulus strength was included as a covariate. Before analysis of covariance (ANCOVA), data for cone photoreceptor and glial amplitudes were analyzed without log-transformation, whereas PSD integrals of OPs were log-transformed to better satisfy the assumptions of linearity, normality, and variance homogeneity. A one-way ANCOVA, followed by Holm-Sidak post hoc test, was then performed to assess statistically significant differences in the stimulus–dependence of the study parameters among the three groups.

Statistical significance was assessed using IBM SPSS Statistics (SPSS Inc., Chicago, IL, USA). In figures, *P* values are given above comparison brackets, or, for post hoc test results, within the corresponding boxes, with values <10^−3^ rounded up. To reduce the risk of Type I errors (or false-positives), *P* values were adjusted by the Bonferroni (for Kruskal–Wallis test) or Sidak (for ANOVA and ANCOVA) correction for multiple tests. Throughout the text (*n*) stands for experimental group size.

## Results

In this study, we investigated the impact of CO_2_ overdose euthanasia on mouse retinal physiology using tERG. Gradual-fill CO_2_ overdose was applied at volumetric displacement rates of 30% (slow) and 60% per minute (fast), and results were compared with those from cervical dislocation.

The tERG response is generated from radially oriented retinal cell types and comprises the sum of negative and positive components that originate from different stages of visual information processing overlapping in time. To assess potential effects of CO_2_ overdose euthanasia on retinal function, we first analyzed the waves (*a*-wave and *b*-wave) and wavelets (oscillatory potentials) and then isolated light-induced responses from rod and cone photoreceptors, ON-bipolar cells (ON-BC), and Müller glia.

### The Euthanasia Method Does Not Affect a- and b-Waves

Representative examples of dark-adapted tERGs with blocked glial component are shown for the different euthanasia methods in Figures 1A and 1B. For the analysis of *a*- and *b*-waves, OPs were removed by low-pass filtering (see Methods, Fig. 2A, Supplementary Fig. S1).

The amplitudes of *a*-wave (Figs. 1C, 1D) and *b*-wave (Figs. 1E, 1F) were similar across all three study groups. The peak *a*- and *b*-wave amplitudes were 0.70 ± 0.11 and 1.30 ± 0.22 mV for cervical dislocation, 0.72 ± 0.06 and 1.24 ± 0.23 mV for slow CO_2_ overdose (30%/min), and 0.74 ± 0.12, and 1.35 ± 0.24 mV for fast CO_2_ overdose (60%/min), respectively (Figs. 1D, 1F).

The *b*-to-*a* wave amplitude ratio indicates how effectively photoreceptors drive second-order retinal cells. At rod-dominated, dim luminance levels (<1000 R*/rod), *b*-to-*a* wave ratios did not differ significantly between the three study groups, though the ratio tended to be smaller after slow CO_2_ overdose (Fig. 1G). At stronger flash strengths (>1000 R*/rod), the ratios were nearly overlapping across the study groups (mean ratios for >1000 R*/rod were 1.90 ± 0.19 for cervical dislocation, 1.78 ± 0.26 for slow CO_2_ overdose and 1.98 ± 0.47 for fast CO_2_ overdose). Thus inner retinal function dominated by rod pathways and mixed rod and cone pathways was not affected by CO_2_ overdose.

Latency values were defined as the time interval between the flash onset and when the responses reached 10% of their maximum. The latency values were similar across the three study groups for *a*-wave (for the brightest flash mean latency values were 6.9 ± 0.3 ms for cervical dislocation, and 6.6 ± 0.3 ms and 6.8 ± 0.3 ms for CO_2_ overdose with 30% and 60% flow rates, respectively, Fig. 1H) and *b*-wave (for the brightest flash mean latency values were 26.8 ± 1.6 ms for cervical dislocation, 26.2 ± 1.1 ms for slow CO_2_ overdose and 28.0 ± 1.4 ms for fast CO_2_ overdose, Fig. 1I).

### CO_2_ Overdose Euthanasia Alters the Properties of Oscillatory Potentials

OPs are high-frequency, low-amplitude waveforms superimposed on the rising phase of the *b*-wave. OPs are thought to originate from synaptic activity within inhibitory feedback circuits in the inner retina, primarily driven by amacrine cells, and are sensitive indicators of metabolic or vascular dysfunction in the retina.^23^^,^^24^

OPs have a significantly higher dominant frequency than the *a*- and *b*-waves. Using a frequency filtering approach, the tERG waveform can be separated into an OP-free tERG trace and isolated OPs. The procedure of OP isolation is illustrated in Figures 2A and 2B (see also Methods and Supplementary Fig. S1).

To compare cumulative properties of OPs between the study groups, the integrals of PSD were computed over the 80–200 Hz frequency range. The PSD integrals at sub-saturating stimuli demonstrated a clear growth after CO_2_ overdose compared to cervical dislocation (Figs. 2C, 2D). A one-way ANCOVA, followed by Holm-Sidak post hoc test, was then performed to assess differences between the study groups over linear range of stimulus – PSD integral relationship (2.3–40 R*/rod; see Methods). Groups euthanized by slow or fast CO_2_ overdose exhibited significantly higher PSD integrals than the cervical dislocation group (both *P* < 10^−3^, Holm-Sidak test; Fig. 2D).

The dominant PSD frequency exhibited a positive frequency shift in mice euthanized by slow CO_2_ overdose (Figs. 2C, 2E). At bright light levels (≥40 R*/rod), dominant frequencies were statistically different across all groups (all *P* < 10^−3^; Holm-Sidak test; Fig. 2E). The median dominant frequency, estimated for the stimulus strength range of 40-8500 R*/rod, was ∼10% higher for slow CO_2_ overdose (139.8 (135.6–141.0) Hz) than for cervical dislocation (125.5 (117.2–134.2) Hz; *P* < 0.001), and ∼6% higher than for fast CO_2_ overdose (131.3 (125.8–135.6) Hz; *P* = 0.010, Dunn's test; Fig. 2F). Such a shift in the OP peak frequency after slow CO_2_ overdose may reflect altered inner retina neural processing.

Averaged isolated OPs at six stimulus strengths are shown in Figure 2G for mice euthanized using the three different methods. Here, we compared only four wavelets—OP1, OP2, OP3, and OP4—with OP2 and OP3 exhibiting the highest amplitude. Although the group-averaged OPs appeared largest in mice euthanized by slow CO_2_ overdose, this effect was due to increased synchronization of OPs (Supplementary Fig. S2A, see also error bars in Fig. 2E). Overall, individual OP1–OP4 amplitudes did not differ significantly between groups, except for higher OP3 amplitudes in the fast CO_2_ overdose group compared with cervical dislocation (*P* = 0.019, Holm-Sidak test; Supplementary Fig. S2B).

In agreement with changes in dominant frequency (Figs. 2E, 2F) and group-averaged OP waveforms (Fig. 2G), ANCOVA with post hoc test revealed a decrease in time-to-peak of individual OPs after slow CO_2_ overdose compared with two other methods. Acceleration in OP wavelets was already significant starting from OP1 and appeared to progress to the later OPs (for OP1-OP4 cervical dislocation vs. slow CO_2_ overdose: *P* < 10^−3^, 0.028, < 10^−3^, < 10^−3^; and slow vs. fast CO_2_ overdose: *P* = 0.002, < 10^−3^, < 10^−3^, < 10^−3^; Holm-Sidak test, Supplementary Fig. S2C).

### Rod Photoreceptor Component Is Unaffected by Euthanasia Method

Application of the synaptic blocker DL-AP4, a selective mGluR agonist, eliminates responses from ON-bipolar cells and isolates the photoreceptor component in the tERG signal when Ba^2+^ is present to block the glial component (Fig. 3A, B). Figures 3A–C show examples of such pharmacological isolation of the photoreceptor and ON-bipolar cell components.

Group-averaged photoreceptor component responses from mice euthanized using the three different methods are shown in Figure 3D. The stimulus–response amplitude curves of the photoreceptor component showed no significant differences between the three groups, indicating similar rod photoreceptor sensitivity (Fig. 3E). The saturated response amplitudes were also similar across groups (0.67 ± 0.12 mV for cervical dislocation, 0.67 ± 0.10 mV and 0.72 ± 0.14 mV for CO_2_ overdose with gradual chamber filling at 30%/min and 60%/min volumetric flow rates; Fig. 3F). There were no significant differences in 10% latencies between the groups (Fig. 3G).

The Lamb and Pugh model^21^ was fitted (Equation 1) to 3-4 sub-saturated responses, as shown in Fig. 3H (see Methods) to determine the phototransduction activation constant *A* and a time delay, *t_d_* (5.4 ± 0.7 ms for cervical dislocation, 5.3 ± 0.9 and 5.8 ± 0.8 ms the slow and fast CO_2_ overdose groups, respectively). The activation constants were not significantly different being 10.9 (9.0–12.8) s^−2^ for cervical dislocation, 11.9 (11.0–12.1) s^−2^ for fast CO_2_ overdose and 11.0 (9.4–12.6) s^−2^ for slow CO_2_ overdose (Fig. 3I).

Dominant time constants of saturated flash response recovery (τ_*D*_) were calculated using Equation 2 (see Methods) from the Pepperberg plots (Fig. 3J), assuming that the duration of response saturation increases linearly with the natural logarithm of flash strength.^22^ The slope of this relationship yields the time constant τ_*D*_, which reflects the rate-limiting kinetic step in phototransduction shutoff. The values of τ_*D*_ were 142 ± 13 ms for the cervical dislocation group, 136 ± 6 ms and 136 ± 7 ms for the slow and fast CO_2_ overdose groups, respectively (Fig. 3K).

### Cone Response Amplitudes Decrease After CO_2_ Overdose

**Figure 4.**
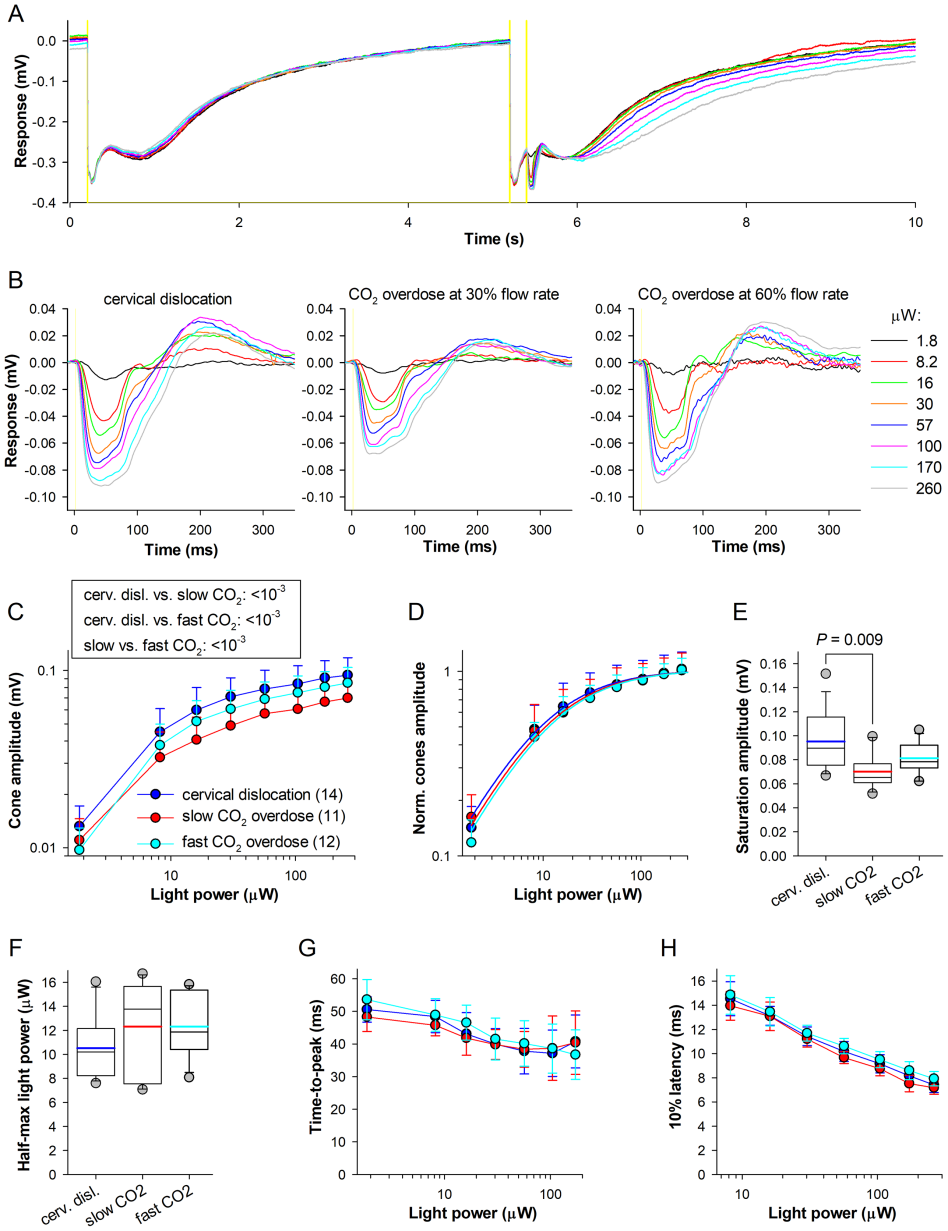
**Cone photoreceptor tERG component.** **(A)** Responses to double flash protocol. The 10-second recording protocol consisted of two parts: the first 5 s included a single 4 ms rod-saturating flash, whereas the second five seconds contained two flashes (*double flash*): a 4 ms rod-saturating flash followed by 2 ms flashes of increasing strengths. Light flashes are indicated with *yellow shading*, rod saturating flash strength was 22100 R*/rod, light flashes for cones were 1.8–260 µW. Cone responses were isolated by subtracting the first half of the recording from the second half. **(B**) Examples of cone responses from the mice euthanized by cervical dislocation (*left*) and CO_2_ overdose (*center, right*). **(C**, **D)** Stimulus-response relationships of cone amplitudes **(C)** and normalized cone amplitudes with Michaelis equation fits **(D)** for the three experimental groups. **(E)** Saturated response amplitudes obtained from fitting. **(F)** Half-maximal light power obtained from fitting for three groups. **(G, H)** Time-to-peak **(G)** and 10% latencies **(H)** as functions of light power.

To isolate dark-adapted mouse cone photoresponses, a double-flash protocol was used as previously described,^19^ in which the first strong flash (∼22100 R*/rod) saturated the rods, and a second flash, presented 200 ms later, was introduced to stimulate the cones (Fig. 4A). Examples of cone responses for mice euthanized using the cervical dislocation and the gradual-fill CO_2_ overdoses are shown in Figure 4B. The stimulus – response amplitude curves showed decreased amplitude for mice euthanized with CO_2_ (Fig. 4C). ANCOVA with post hoc test revealed a significant reduction in the amplitude between study groups (all *P* ≤ 10^−3^, Holm-Sidak test). Saturated response amplitude and half-maximal light power were estimated from the fitting of the Michaelis equation to stimulus – response amplitude curves using Equation 3 (see Methods; Fig. 4D). The saturated cone amplitude was ∼26% smaller in the slow CO_2_ euthanasia group (0.065 (0.061–0.077) mV) than in cervical dislocation group (0.089 (0.074–0.114) mV); *P* = 0.009, Dunn's test; Fig. 4E). Amplitudes of saturated cone responses in mice euthanized by fast CO_2_ overdose (0.078 (0.073–0.091) mV; Fig. 4E) did not differ significantly from the two other groups, as assessed by the Kruskal–Wallis test. Cone sensitivity (estimated as a reciprocal of the half-maximal light power; Figs. 4D, 4F), time-to-peak (Fig. 4G), and 10% latencies (Fig. 4H) were also not affected by CO_2_ euthanasia.

### Rod ON-Bipolar Cell Component Is Not Influenced by CO_2_ Overdose

**Figure 5.**
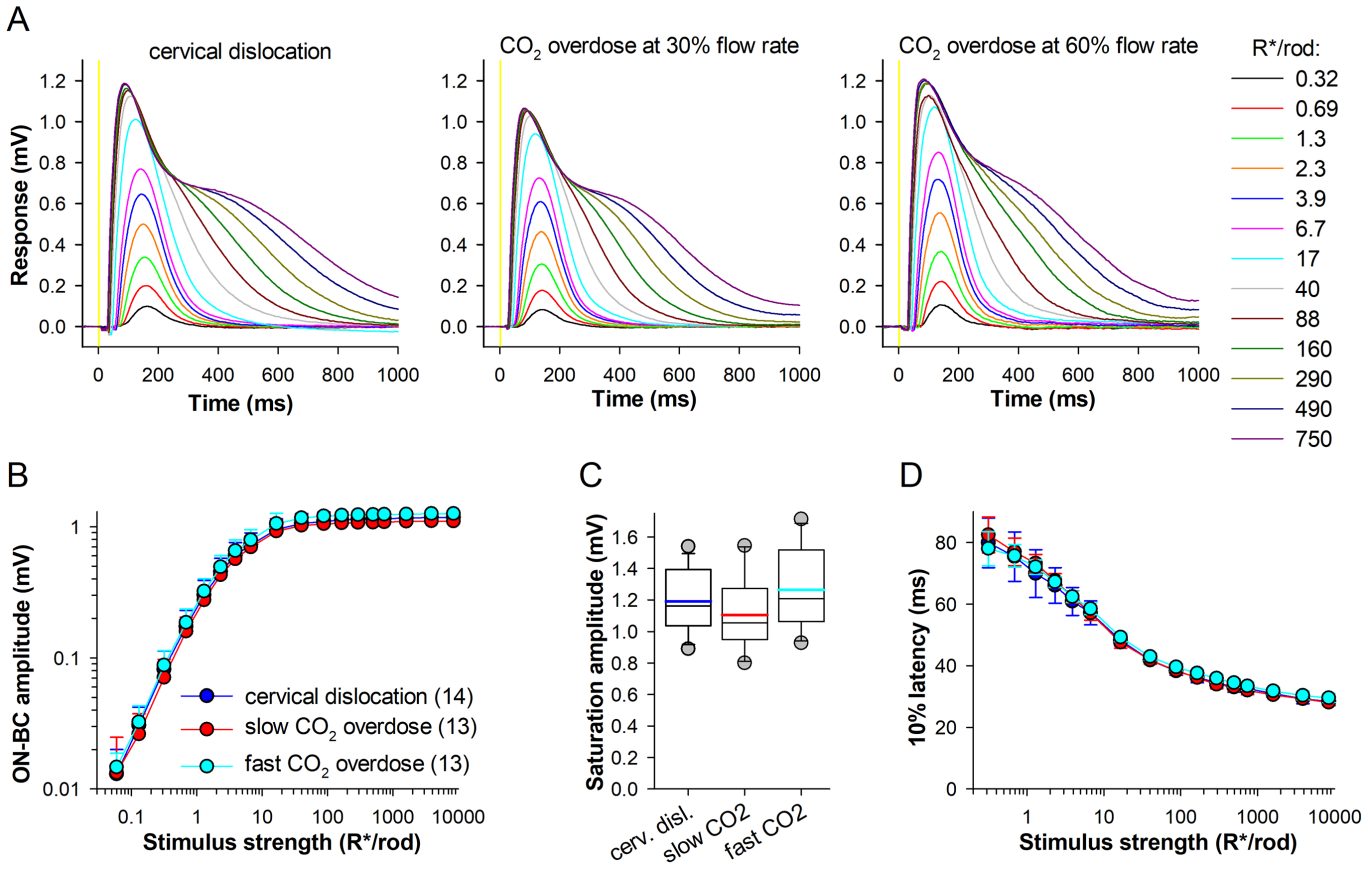
**Rod ON-bipolar cells tERG component.** **(A)** Representative examples of isolated rod ON-bipolar cell components from mice euthanized by cervical dislocation (*left*) and slow and fast CO_2_ overdose (*center, right*); all traces were low-pass filtered to remove OPs. **(B**, **C)** Stimulus-response relationships **(B)** and its saturated response amplitudes **(C)** for the ON-bipolar cell component across the three groups. **(D)** The 10% latencies as functions of stimulus strength for three groups.

Figure 5A shows the isolated rod ON-bipolar cell component recorded from mice euthanized by cervical dislocation and by gradual-fill CO_2_ overdose. The stimulus–response amplitude curves, and thus the sensitivity of ON-bipolar cells, were similar across the groups (Fig. 5B). Likewise, the saturated response amplitude of the ON-bipolar cell component showed no significant differences between groups (1.16 (1.04–1.40) mV for cervical dislocation; 1.06 (0.95–1.28) mV and 1.21 (1.06–1.52) mV for CO_2_ overdose with 30% and 60% flow rates, respectively; Fig. 5C). Latencies were also similar across the three groups (Fig. 5D). These results indicated that carbon dioxide euthanasia does not affect rod phototransduction or synaptic transmission from rod photoreceptors to ON-bipolar cells.

### Müller Cell Component Is Altered by the Euthanasia Method

**Figure 6.**
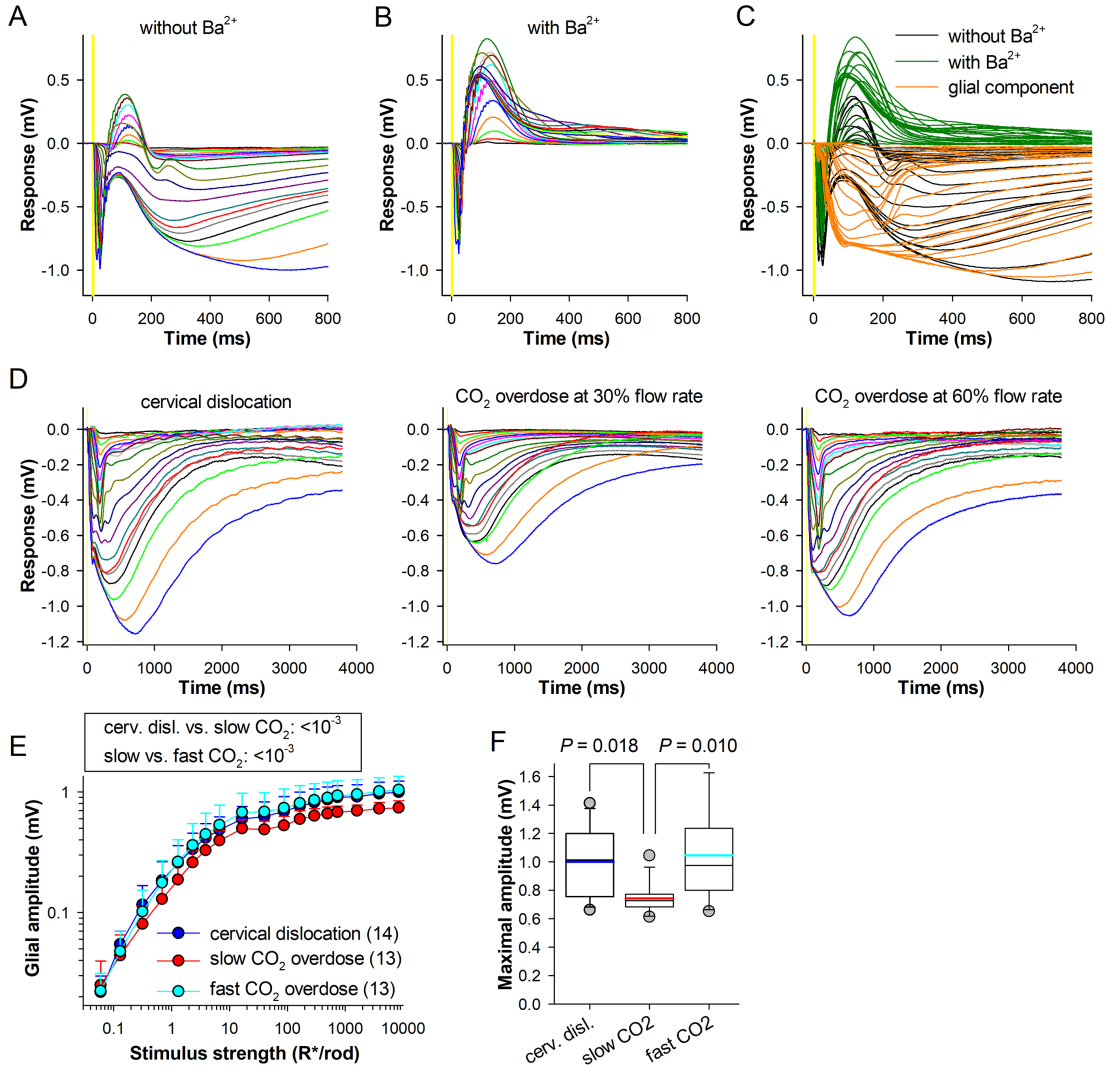
**Glial tERG component.** **(A**, **B)** TERG recordings from a wild-type isolated mouse retina before **(A)** and during application of BaCl_2_ **(B)**; flashes of increasing strengths are from 0.06 to 8500 R*/rod. **(C)** Subtraction of **A** (*black traces*) from **B** (*green*) gives isolated glial responses (*orange*, OPs were removed from traces in **C** and **D** by low-pass filtering). **(D)** Examples of isolated glial responses from WT mice euthanized by cervical dislocation (*left*) and slow (*center*) and fast CO_2_ overdose (*right*). **(E)** Stimulus-response relationships of glial response amplitudes in the three groups. **(F)** Maximal amplitudes of glial tERG component were significantly different for slow CO_2_ overdose group.

The tERG responses were recorded from isolated wild-type mouse retinas during perfusion with Ames’ medium without and with 100 µM of BaCl_2_ (Figs. 6A, 6B). Ba^2+^ blocks K^+^ channels located mainly at the Müller cell endfeet and suppresses the glial component of the tERG waveform. The glial component was then obtained by subtracting the tERG with Ba^2+^ from the control tERG (Fig. 6C).

Examples of isolated Müller cell responses for the three different euthanasia methods are shown in Figure 6D. The maximal amplitude of the glial component was ∼25%–27% smaller in mice euthanized by slow CO_2_ overdose (0.73 (0.68–0.77) mV) in comparison to both cervical dislocation (1.01 (0.76–1.20) mV; *P* = 0.018) and fast CO_2_ overdose (0.97 (0.80–1.23) mV; *P* = 0.010, Dunn's test; Figs. 6E, 6F). At sub-saturating light levels, ANCOVA with post hoc test revealed a similar but stronger significance (both *P* < 10^−3^, Holm-Sidak test). Furthermore, the amplitude of the glial component was less variable in mice euthanized by slow CO_2_ overdose.

## Discussion

In the present study, we found that CO_2_ overdose alters light-evoked responses of retinal cells, specifically those expressing carbonic anhydrase (CA) (Figs. 2, 4, 6). At least three isoforms of carbonic anhydrase, intracellular CA II, transmembrane CA XIV and intracellular CA C, are present in the adult mammalian retina.^25^^–^^30^ These enzymes catalyze the reversible hydration of CO_2_ to produce H^+^ and bicarbonate, and they play a key role in pH regulation within the retina. CA II is expressed in Müller cells, in most cone photoreceptors (possibly excluding blue-sensitive cones^31^), and in a subset of amacrine cells.^25^^,^^29^^,^^32^ CA XIV is found on both apical and basolateral membranes of the retinal pigment epithelium and on specific membrane domains of Müller cells and astrocytes.^25^^,^^26^ During development, CA C is initially present ubiquitously but in adults becomes restricted to Müller glia and a subset of amacrine cells.^27^ In contrast, neuronal cell types such as rod photoreceptors, bipolar cells, and ganglion cells do not express CA at appreciable levels,^25^^,^^29^^,^^31^ likely relying on neighboring Müller glia and the retinal pigment epithelium to buffer and remove H^+^ from the retinal extracellular space. Among the retinal cells, Müller cells contain the highest levels of CA.^26^^,^^33^

During CO_2_ euthanasia, the partial pressure of CO_2_ can rise two- to threefold. At high CO_2_ tissue levels, the hydration of CO_2_ into H⁺ and HCO_3_⁻ accelerates, increasing the H^+^ load.  Carbonic anhydrases catalyze the CO_2_ hydration reactions, and CO_2_ overdose-induced intracellular H⁺ production may be very high in CA-expressing cells, causing a quick drop in intracellular pH. Acidification has many detrimental effects; for example, it increases metabolic burden, alters the activity of voltage-gated and ligand-gated ion channels (typically in a reversible manner), and can trigger destructive irreversible downstream pathways through proteolysis and excitotoxic mechanisms.^34^^,^^35^

### Effects of CO_2_ Overdose on Rod Phototransduction and Synaptic Transmission Are Reversible, if Present

Although an immediate drop in extracellular pH to 6.0 was reported for brain tissue after CO_2_ overdose,^17^ we did not find any impact of CO_2_ overdose on rod and ON-bipolar cell responses, indicating that rod photoreceptor and ON-bipolar cell function, as well as synaptic transmission from photoreceptors to ON-bipolar cells, were not affected by CO_2_ overdose (Figs. 1, 3, 5). Previous studies have demonstrated that phototransduction tolerates extracellular pH fluctuations well: when perfusate pH was reduced from 7.3 to 6.5, the rod response amplitudes remained unchanged in amphibians.^36^ Extracellular acidification to pH ∼4.8 suppresses ≥50% of the saturated amphibian rod response amplitude, prolongs the plateau phase, and slows recovery from a saturating flash, whereas acidification to pH ∼5.6 produces only ∼10% dark current suppression.^37^^,^^38^ To our knowledge, however, no similar studies on mammalian phototransduction have been reported. Instead, synaptic transmission in the retina is more sensitive than phototransduction to acidification: lowering pH from 7.4 to 7.1 caused ∼20%–30% decrease in the *b*-wave amplitude in the isolated perfused cat eye.^39^ In isolated mouse retina, a pH drop from 7.4 to 6.8 led to a rapid ∼80% reduction in *b*-wave amplitude within just five minutes.^40^ However, the effects of acidosis were largely reversible, as *b*-wave amplitude fully recovered under normal perfusion conditions, even after 60 minutes’ incubation in the low pH solution.^40^

Holtzmann et al.^17^ reported that after the CO_2_ overdose, pH did not recover during their five-minute measurements from the opened skull. In our study, CO_2_-induced euthanasia took ∼10 minutes with 30 vol-%/min flow rate and ∼8.5 minutes with 60 vol-%/min rate, followed by removal of both eyes and dissection of the retinas lasting ∼10 minutes. However, during and after dissection, the retinal tissues were in bicarbonate-buffered Ames’ solution, bubbled with carbogen to maintain a pH of ∼7.3. Perfusion and recording on the first retina began 15 to 20 minutes after euthanasia, most likely allowing rod and ON-bipolar cells to almost fully recover from the CO_2_-induced acidosis. This is further supported by the absence of differences in responses between the first and second retinas from the same animal, despite the second retina having a much longer incubation and therefore more time for pH recovery.

### CO_2_ Overdose Decreases Cone Photoresponse Amplitudes

The prevalence and morphology of the CA-expressing cones in human retinas suggest that these are red- and green-sensitive cones, although opsin identity was not directly confirmed.^31^ Meanwhile, in our study we have recorded from the central retina and specifically stimulated green-sensitive (expressing M opsin and M/S opsin) cones, which, in contrast to UV-sensitive cones, show a relatively small variation in the spatial density within the central region.^41^^,^^42^ Nevertheless, local variations in pure S opsin cone cell density cannot be completely ruled out as contributing factors to variability in the recorded responses under green-light stimulation.

In this context, it is noteworthy that, unlike in rods, the photoresponses in cones were irreversibly smaller after CO_2_ overdose compared with those after cervical dislocation. Both rods and cones contain powerful pH regulation mechanisms (Na^+^/H^+^ exchangers and Na^+^-dependent and -independent HCO_3_^−^/Cl^−^ exchangers) in their plasma membrane, preventing intracellular acidification due to the high metabolic activity of photoreceptor cells.^43^^–^^46^ Inhibition of either of the Na^+^-driven exchanges, Na^+^/H^+^ exchange or Na^+^-dependent HCO_3_^−^/Cl^−^ exchange, leads to intracellular acidification and to a concomitant decrease in photoresponse amplitudes both in rods and cones.^43^^,^^44^ The CO_2_ overdose-induced reduction of photoresponse amplitudes in cones but not in rods suggests that the pH regulation mechanisms can prevent acidification during CO_2_ euthanasia only in rod photoreceptors. A plausible explanation for this difference may be the presence of CA in cones but not in rods: CO_2_ overdose causes much faster H^+^ production in cones, exceeding the capacity of the pH regulation mechanisms to oppose acidification and leading to irreversible damage in cones. Consistent with a prominent role of CA activity in cones, the CA inhibitor acetazolamide reduces the circulating current in cones but not in rods under comparable conditions.^43^^,^^47^^,^^48^

### Müller Cells Are Vulnerable to Retinal Homeostasis Disruption

In the retina, Müller cells are often the first responders to minor disturbances in retinal homeostasis.^49^ In vitro, brain glial cells begin to swell within one to three minutes in response to a pH drop to 6.8 or lower.^50^ Respiratory acidosis after CO_2_ overdose leads to increased mechanical stiffness of the gray matter tissue in rat brain.^17^ Müller cells, and likewise other glial cells in various tissues, contribute to increased retinal stiffness by undergoing reactive gliosis, cytoskeletal remodeling, and secretion of extracellular matrix components (Prieto-Lopez et al., 2024). Analogous effects may be expected for retinal layers that are rich in cell bodies and synapses and thus resemble gray matter.

In the present study, we observed a substantial reduction in the glial response after the slower CO_2_ overdose (Figs. 6D–F). The neural activity-induced Müller cell response is largely generated by potassium currents through inwardly rectifying (Kir) channels that are inhibited by intracellular acidification,^51^^–^^53^ consistent with the hypothesis that the decline in Müller cell responses might be caused by intracellular acidosis. However, the glial responses remained practically unaffected by the faster CO_2_ overdose, suggesting that glial hypertrophy and gliosis might require a longer CO_2_ exposure, possibly being a combined effect of longer-lasting acidosis and hypoxia.

### Combined CO_2_-Induced Acidosis and Hypoxia Can Lower the Threshold for Irreversible Cellular Damage

CO_2_-induced euthanasia involves not only acidosis but also hypoxia. The high metabolism of the retina results in oxygen consumption rates that exceed those of brain tissue, and oxygen deprivation rapidly shifts metabolism toward glycolysis, leading to accumulation of lactate and H^+^ ions. Hypoxia alone (without excessive CO_2_) causes a rapid decline in retinal signaling. The *b*-wave, reflecting ON-bipolar cell function, is a highly sensitive marker of hypoxia and exhibits progressively irreversible loss after tens of minutes of anoxia.^40^ In the present study, however, we did not observe a reduction in *b*-wave amplitude after ∼10 minutes of CO_2_ overdose, likely due to the immediate dissection and subsequent perfusion with oxygenated, bicarbonate-buffered Ames’ solution, which restored near-physiological conditions ex vivo. Therefore the threshold for irreversible cellular damage was unlikely to have been reached by hypoxia alone in our experiments. Instead, inhalation of concentrated CO_2_ produces tissue acidosis in two ways, through hypoxia and through hydrolysis of excessive CO_2_, and cellular vulnerability is strengthened under combined hypoxic–acidotic stress.^54^^,^^55^ Consistent with this, adding ischemia-like cofactors such as hypoxia shifts lethality to shorter exposures and higher pH: neither hypoxia alone nor acidosis alone (25 minutes at pH 6.6) induces astrocyte death, whereas their combination produces ∼20% astrocyte loss.^55^ Furthermore, exposure to elevated CO_2_ for as little as five minutes markedly accelerates the degradation of ATP and GTP into purine metabolites in the retina, resulting in metabolic arrest and extensive nucleotide breakdown.^56^ The resulting accumulation of purine metabolites not only reflects impaired energy homeostasis but can also exacerbate oxidative stress through xanthine oxidase–mediated pathways and by promoting reactive oxygen species generation. Such metabolic disturbances compromise both neuronal and glial viability and are likely to contribute to secondary degenerative processes in the hypoxic retina.

### Oscillatory Potentials as Functional Biomarkers of Inner Retinal Activity and the Possible Role of Dopamine

OPs are generated within the inner retina and are primarily mediated by amacrine cells. OPs are widely regarded as functional biomarkers of inner retinal activity. They are usually observed in vivo, but have occasionally been reported ex vivo, although with reduced amplitudes.^57^^,^^58^ In this work, we report prominent OPs, ∼0.23–0.27 mV in amplitude (∼18%–20% of *b*-wave amplitude), indicating that the inner retina physiology in our ex vivo conditions is close to that in in vivo studies.

Abnormalities in OPs have been reported in various models of retinal degeneration, making them valuable for both basic and translational research. For example, in diabetic subjects, dark-adapted OPs (OP1–OP4) exhibit significantly delayed time-to-peaks even before clinically detectable retinopathy.^59^^–^^61^ In the present study, however, we did not observe delayed OPs; instead, the dominant frequency was shifted towards higher frequencies after CO_2_ overdose (Figs. 2E, 2F), indicating a faster, more temporally compact OP waveform. Consistent with this interpretation, statistically significant acceleration in individual OP wavelets was observed and appeared to progress to the later OPs (Supplementary Fig. S2C).

Although we did not try to study the causes of the OPs acceleration and increase in PSD, a clue might come from previous studies on retinal dopamine. A decreased level of retinal dopamine was reported after CO_2_ overdose,^18^ and dopamine deficiency was correlated with increased delays in OPs mediated through rod pathways.^61^^,^^62^ Furthermore, administration of the dopamine precursor L-DOPA accelerated OPs,^63^ suggesting that changes in dopamine levels can modulate oscillatory potentials. However, studies on the effects of dopamine and dopaminergic drugs on OPs have reported conflicting results. In rabbits, D1 receptor blockade with SCH-23390 selectively reduced OP2 amplitude, whereas the D1 agonist SKF-38393 had little effect.^64^ In the mudpuppy retina, either haloperidol (a D2 antagonist) or dopamine itself produced a selective decrease of OPs.^23^ Increased central dopaminergic transmission (in schizophrenia) has been associated with more variable OPs.^65^ Taken together, these findings indicate that dopaminergic modulation of OPs is evident, but the direction and magnitude depend on the experimental model, receptor subtype, and disease context.

Interestingly, dopamine depletion by 6-hydroxydopamine (6-OHDA), a neurotoxin that selectively destroys dopaminergic and noradrenergic neurons, significantly enhanced OP amplitudes, an effect reversed by the dopamine agonist apomorphine.^66^ Considering the reported decrease in retinal dopamine after CO_2_ euthanasia,^18^ this could provide a plausible explanation for the observed increase in PSD integrals (Fig. 2D). However, because 6-OHDA depletes both dopamine and noradrenaline, the specific effects of dopamine loss on OPs remain unclear. Acceleration and amplification of OPs after CO_2_ overdose suggest altered processing in inner retinal circuits. These changes may be compensatory or pathological, but because of the composite origin of OPs, the precise interpretation remains challenging and requires additional investigations.

Retinal dopamine release is higher during the subjective day and is under circadian control. In addition to modulating inner-retinal inhibitory circuitry, dopamine dynamically regulates gap junction coupling in multiple retinal networks, including rod-cone, rod-rod, cone-cone, AII amacrine-AII amacrine coupling, AII amacrine-ON cone bipolar coupling, and horizontal cell coupling.^67^ Nevertheless, in our dataset, we did not observe systematic differences in tERG waves/components or OPs properties between subjective day and subjective night recordings, suggesting that circadian variation in dopaminergic tone was not a major contributor to the effects reported here.

## Supplementary Material

Supplement 1
