# Supplementary material for: Carbon Dioxide Euthanasia Selectively Affects Physiology of Murine Retinal Cells, Implicating Carbonic Anhydrase-Expressing Cell Populations
Source: Invest Ophthalmol Vis Sci. 2026 Mar 31;67(3):61. doi: 10.1167/iovs.67.3.61 (PMC13044628; doi:10.1167/iovs.67.3.61)
Supplement: Supplement 1 [file iovs-67-3-61_s001.pdf]

Carbon dioxide euthanasia selectively affects physiology of murine retinal cells, implicating carbonic anhydrase-expressing cell populations: Supplementary material

Irina Ignatova[1], Ari Koskelainen[1]

[1] Department of Neuroscience and Biomedical Engineering, School of Science, Aalto University, Finland

Corresponding author: Irina Ignatova; e-mail: irina.ignatova@aalto.fi

ORCID: Irina Ignatova 0000-0002-9861-2640; Ari Koskelainen 0000-0003-3842-8283

Parent Article DOI: 10.1167/iovs.0.0.45880

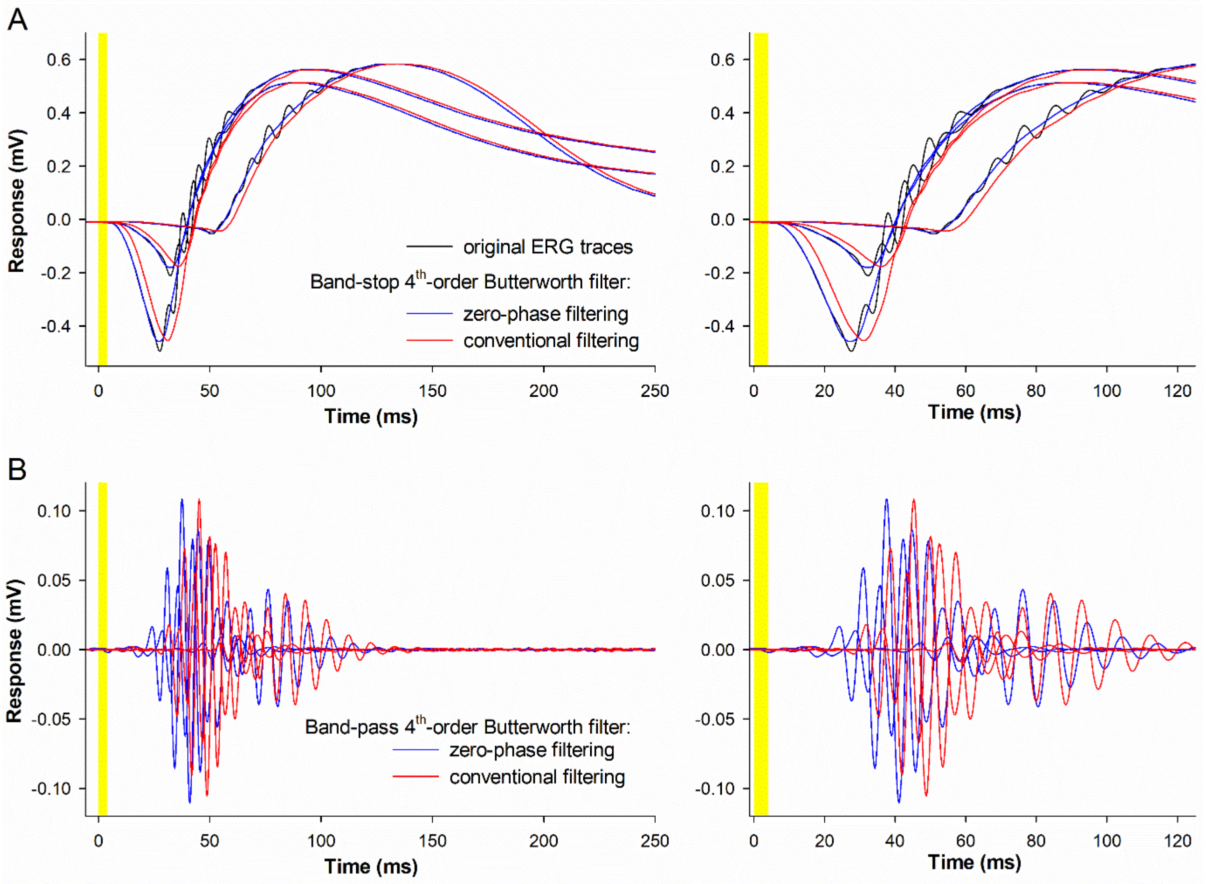

Supplementary Figure S1. Frequency filtering of tERG waveforms. (A, B) The tERG responses (*black traces*) were separated into OP-free tERG traces **(A)** and isolated OPs **(B)** using a zero-phase 4th-order Butterworth filter (*blue traces*). A conventional 4th-order Butterworth filter (*red traces*) introduces frequency-dependent phase delay. On the left and right, the same traces are shown at different time scales.

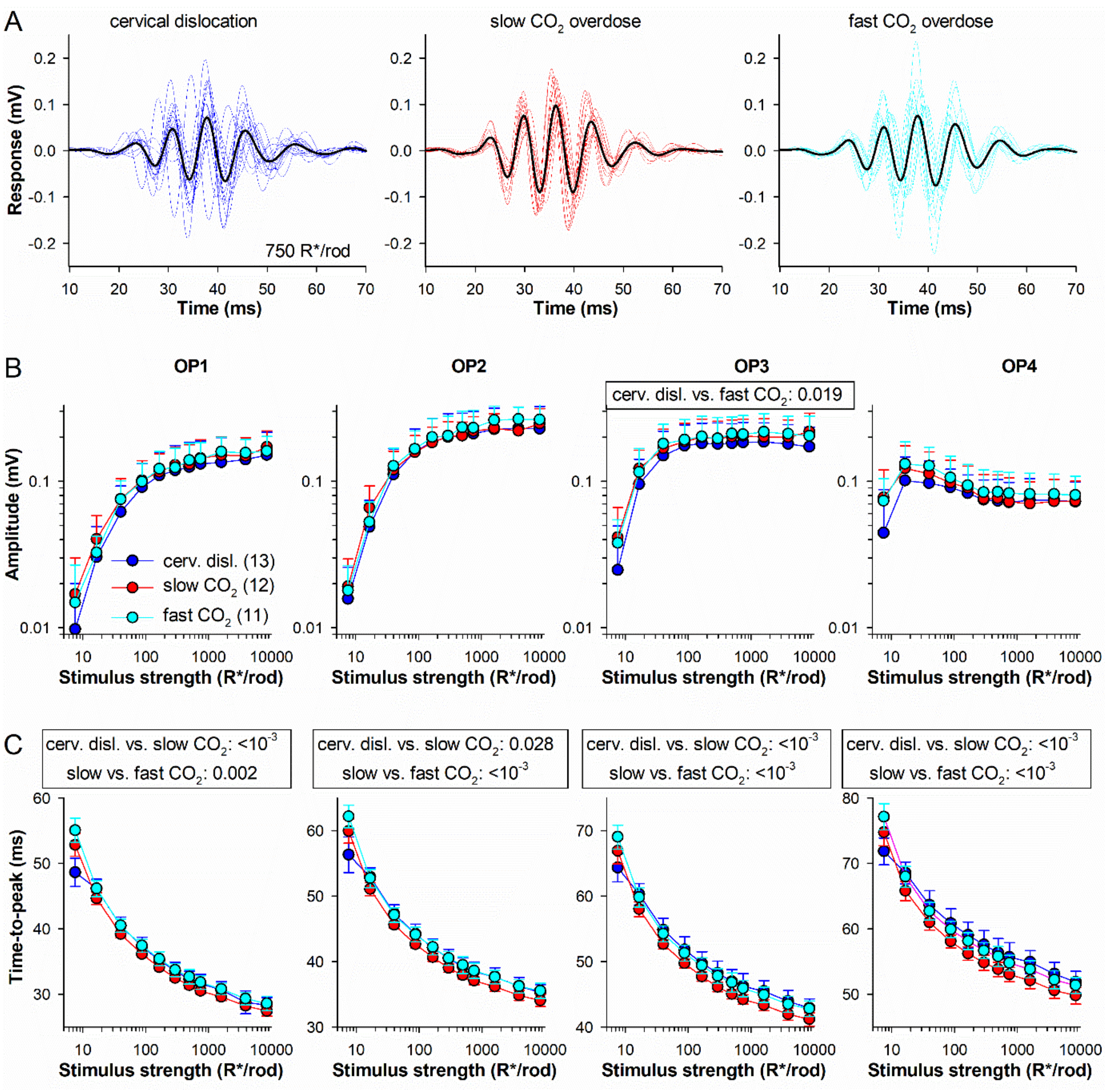

Supplementary Figure S2. Individual oscillatory potentials. (A) Individual and group-averaged (*black line*) OPs for mice euthanized using three different methods at ~740 R*/rod. **(B, C)** Stimulus-response relationships of the amplitudes **(B)** and time-to-peak **(C)** of individual OP1-OP4 for the three study groups.
